# Vulnerability, impact and adaptation strategies of female farmers to climate variability

**DOI:** 10.4102/jamba.v14i1.1302

**Published:** 2022-09-15

**Authors:** Siphosethu Dibakoane, Pakama Siyongwana, Ayanda N. Shabalala

**Affiliations:** 1 Faculty of Agriculture and Natural Sciences, University of Mpumalanga, Mbombela, South Africa

**Keywords:** females, constraints, climate variability, adaptation strategies, agrarian-based livelihoods

## Abstract

In Africa, agriculture, particularly crop production, is a vital livelihood practice for women, who provide a larger proportion of the labour force. However, the high reliance on rain-fed agriculture, coupled with other socio-economic constraints, exposes female farmers to climatic risks. This paper investigates the participation of women in crop production, key challenges and their coping strategies for climatic disturbances. Drawing on the experiences of female farmers of Thaba Chweu Local Municipality (TCLM) in Mpumalanga, South Africa, the study blended qualitative and quantitative approaches to gather data on their vulnerability and adaptation strategies to climatic shocks. A questionnaire administered through face-to-face interaction and online surveys was the main instrument used to obtain data. This study revealed diverse challenges faced by female farmers in the form of high susceptibility to climatic disruptions, limited funding and gaps in accessing agricultural inputs and equipment (machinery, seeds and fertilisers) and pests. The effects of climate variability manifest in low crop outputs and inferior yields, food insecurity and loss of revenue. The most preferred coping strategies are changing planting and harvesting dates, followed by eating less food, looking for jobs and crop rotation. Although the main source of support comes from both family and government, the majority of the female farmers do not use modern scientific-based and input-intensive agricultural coping strategies such as the use of irrigation systems because of lack of livelihood assets and lower literacy levels.

## Introduction

Agricultural activities, particularly crop production, are vital livelihood support systems for farmers in Africa (Njieassam 2019:1–33). These farmers rely on agricultural activities for employment opportunities, generating income and other socio-economic needs. The majority of female farmers in Africa are vulnerable to climate variability because of the over-reliance on rain-fed agriculture and other nonclimatic barriers (Molua 2011:21–35). Studies by Akinseye, Ajayi and Oladitan (2013:91–98) and Recha, Makokha and Shisanya (2017:1–8) demonstrated that alterations in rainfall patterns retard early crop development, which subsequently results in reduced yields. Existing evidence reveals that female farmers are vulnerable to climate change because of limited access to agricultural extension and training services, farming land, funding and markets (Adeoti, Coster & Akanni 2016:1–16; Ugwu 2019). Although some female farmers have resorted to indigenous knowledge to cope with climate variability (Abeka et al. 2012:8–51), their coping capacity tends to be hindered because of the inability to change cropping cycles or procure high-yielding varieties during seasons of high rainfall variability. Hence, this study aims to provide an empirical report on challenges faced by female crop farmers as well as their vulnerability to climate variability in the Thaba Chweu Local Municipality (TCLM), South Africa.

In the TCLM, crop production has been identified as a potential stimulator of economic growth as a result of the developing domestic and global market, population growth and increased demand for food. Changing climatic conditions have threatened the economic potential of crop production in the municipality as a result of altered rainfall patterns and increased temperatures. Moreover, crop production serves as a livelihood support system for most women in the municipality. Azad, Hossain and Nasreen (2013:190–199) and Nnadi et al. (2019) conducted studies that revealed that female farmers in Bangladesh and Nigeria are mostly vulnerable to complete crop failure during episodes of heavy precipitation. This resulted in a significant decrease in the amount of money that was derived from crop production. Similarly, Ezemonye (2015:109–116) reported that female farmers were more vulnerable to income loss because of flooding compared to those who served in other sectors of the economy. Their vulnerability is exacerbated by lack of private property, high-yielding varieties and inputs, training and extension services, credit and access to markets and the inability to make decisions to determine their own livelihoods (Ugwu 2019). Akinola (2018) noted that women own less than 1% of the land in Africa. Thus, they will not be able to alter their cropping cycles to respond to evolving climatic conditions, as most of the land is owned by their husbands, resulting in less decision-making power. This inhibits their ability to respond effectively to flooding and harsh climatic conditions. In addition, the inability to procure drought-resistant varieties implies that women are not able to produce crops of high quality because most of the crops will be subject to high temperature extremes.

Ubisi et al. (2017:27–38) noted limited information regarding the effects of climate variability on smallholder farmers in South Africa. A study that examines the vulnerability of smallholder farmers to climate extremes and the strategies that are put in place to cope with their impacts becomes very critical. Furthermore, Drucza and Peveri (2018:180–189) noted that scientific agricultural literature has ignored the views of women and privileged that of men (patriarchy), yet female farmers constitute a great proportion of the workforce in agricultural activities such as crop production. Therefore, there seems to be limited knowledge in the literature regarding the constraints that women face in crop farming in the context of a changing and variable climate system. This study seeks to fill this knowledge gap by examining the vulnerabilities and coping strategies of female crop farmers in the TCLM. The aim of the study is to provide an empirical report on the key challenges faced by women crop farmers in TCLM. Specifically, the study’s objectives were to identify institutional, societal, cultural and economic constraints faced by female crop farmers; determine the impact of climate variability on the livelihood support systems of female crop farmers; and identify adaptation strategies used by female crop farmers in response to challenges they face.

## The theoretical framework

The sustainable livelihood approach (SLA) introduced by Scoones (1998) was chosen as a theoretical framework for this study. The framework has been adapted by Serrat (2017:21–26) to explain how the relationship between people and the environment influences livelihoods. The framework elucidates the potential of people in relation to their capabilities, access to livelihood assets and the ability to influence institutions and identifies the factors that constrain or enhance livelihood opportunities. Furthermore, it acknowledges that households have differentiated access to livelihood assets and improve the intervention of institutions and organisational processes by developing strategies and classifying livelihood assets based on the vulnerability context and the institutional context where they exist (Sati & Vangchhia 2017). Three variables of livelihoods under the SLA framework are assets, policies and institutions and vulnerability contexts (Morse & McNamara 2013). Vulnerability context refers to the disturbance of the well-being of individuals, households and communities to changes in the external environment. This can be in the form of shocks, stresses, trends and the inability to cope with environmental changes (Serrat 2017:21–26). Changes in the external environment are caused by shocks (floods and droughts, pest outbreaks and collapse of markets) and stresses (poor soil conditions, declining water and rainfall levels, depletion of resources and loss of income) (Chambers & Conway 1992). In South Africa, smallholder farmers have experienced changes in their agricultural outputs and productivity because of changes in rainfall patterns and increasing temperature levels (Babugura, Mtshali & Mshali 2010; Ubisi et al. 2017:27–38). These changes have resulted in floods, droughts, declining water and rainfall levels, declining yields and loss of income (Babugura et al. 2010). In particular, the livelihoods of female farmers have been negatively affected by climate-related changes because of loss of income, food insecurity and low crop productivity.

Livelihoods are expected to cope and recover from shocks and maintain their capabilities, assets and activities without depleting the natural resources base. The livelihoods of female farmers are usually less able to cope and recover fully from shocks and stresses because of limited livelihood assets (natural, physical, social and financial capitals), customary laws and limited decision-making powers (Belay et al. 2017:1–13; Lolig et al. 2014:542–553). Myeni et al. (2019:3003) revealed that female farmers in the Free State, South Africa do not have the power to allocate financial resources (capital) despite the possibilities of having them as part of the main working force on the household farm. Scoones (1998) noted that assets can be combined or used for the accumulation of profit to acquire more assets or to cope with the effects of shocks. Therefore, combination of livelihoods is not feasible and may have negative effects on female farmers because of their limited access to livelihood assets.

Access to livelihood assets is determined by institutions which have been identified as being role players in the quest to attain sustainable livelihoods. These structures are designed to implement policies, deliver services and perform tasks that have a bearing on livelihoods and enable stakeholders to exercise their power and determine the manner in which the flow of resources will take place (Serrat 2017:21–26; Scoones 1998). In South Africa, female farmers tend to have limited access to livelihoods because of customary laws and institutional policies and processes enacted by the government (Cousins 2013:73–99; Hart & Aliber 2012:48; Kaarhus et al. 2005:443–482; Rangan & Gilmartin 2002:633–658). Therefore, the state should enact polices that enhance women’s access to livelihoods and reduce their vulnerability to climatic disturbances. This can be done by disaster preparedness, provision of dry season employment through public works and extension services to provide farmers with supplementary knowledge and technology (Chambers & Conway 1992; Morse & McNamara 2013).

It has been suggested that the vulnerability of livelihoods can be mitigated through agricultural intensification and extensification, livelihood diversification and extensification. A study by Ezemonye (2015:109–116) demonstrated that women who were engaged in nonagricultural activities were less vulnerable to climate variability compared to women who were dependent on crop farming. However, most women are not able to mitigate their livelihoods’ vulnerability. This is because of limited migration rights, limited livelihood assets and weak customary laws (Belay et al. 2017:1–13; Lawson et al. 2020:439–452; Lolig et al. 2014:542–553).

Historically, women in Africa were expected to take over agricultural work and ensure the welfare of the family as the men migrated to the city to engage in nonagricultural activities. However, in the modern day, women are allowed to travel because of changes in social beliefs and economics. In South Africa, the migration of women in the postcolonial state has been fuelled by low marital rates, decreasing proportion of women living with men and instability of household income in rural areas (Posel 2006:217–231, 2010:129–141). Migration facilitates the exchange of knowledge, skills and technology, which improves the livelihood strategies of the people by improving the fertility of the land as a result of population reduction. Despite these trends, some authors have argued that the Immigration Act limits migration rights of women in South Africa (Crush & Dodson 2007:436–454); hence, feminist theory becomes a relevant issue in the SLA framework.

The potential livelihood outcomes include improved income, reduced vulnerability, improved food security and sustainable use of resources (Sati & Vangchhia 2017). Livelihood outcomes are aimed at improving the well-being of people and resilience of livelihood systems and can be achieved through proper and equitable allocation and combination of livelihood assets. However, conflict is inevitable because the development process will always favour one group and disadvantage the other group. Livelihood outcomes tend to be gender biased as men tend to be allocated more livelihood assets compared to their female counterparts (Belay et al. 2017:1–13; Lolig et al. 2014:542–553). Hence, more research is warranted to assess the effects of various barriers on the vulnerability of female farmers to climate variability, its effects on their livelihoods and their coping strategies.

## Literature review

### Female farmers’ vulnerability to climate variability

Climate change continues to trigger intensive discourse in gender issues. Galiè et al. (2017:1–8) revealed that women are more involved in farming than their male counterparts. In South Africa, the participation of women in agricultural activities has been suggested to be greater than that of men (Elum, Modise &Marr 2017:246–257). Maponya, Mpandeli and Oduniyi (2013:73–82) reported that the proportion of women (79%) was greater than that of men (29%) in Mpumalanga. Ubisi et al. (2017:27–38) and Maponya and Mpandeli (2012:48–60) confirmed that the proportion of women (54% – 64%) participating in farming activities was greater than that of men (46%) in the Limpopo Province. Female farmers conduct most of the tasks in the farm such as ploughing the fields, removing weeds, monitoring crops and harvesting dates (Galiè et al. 2017:1–8; Nahusenay 2017:144–155). They also bear the unequal responsibility of catering for household food security, harvesting water and fuel for everyday survival (Maponya et al. 2013:73–82).

However, the contributions of female farmers in the agricultural sector are not recognised because they are perceived as being unproductive (Drucza & Peveri 2018:180–189). Furthermore, it has been reported that the drudgery of farming activities and limited access to agricultural inputs tend to affect the participation of female farmers in agricultural activities in Mpumalanga, South Africa (Ajala 2017). Myeni et al. (2019:3003) attributed the limited participation to inadequate decision-making powers, skewed land ownership patterns and cultural ideologies which promote superiority of men over women. In addition, female farmers in South Africa receive less state support (e.g. agricultural extension services) compared to their male counterparts (Hart & Aliber 2012:48).

In most developing countries, including sub-Saharan Africa, customary and statutory laws forbid women from owning land (Chilese & Marirajan 2018: 22–31; Muema et al. 2018). For example, women have limited access to land compared to their male counterparts in South Africa because of customary law (Cousins 2013:73–99; Kaarhus et al. 2005:443–482). In South Africa, land reform policies and programmes such as the Restitution Act, Community Property Association and the Land Reform Pilot Programme have not improved female land ownership rights owing to their lack of participation in land allocation processes (Meer 1997:133–144). Other land reform policies such as land redistribution, restitution and tenure and the recently formed Land Expropriation Without Compensation (LEWC) policy have not been able to address gender-related land ownership inequalities because of their Eurocentric approach and ignorance of the impact of gender in land-related matters (Mubecua & Nojiyeza 2019:7–19). In addition, female famers in South Africa have limited access to credit (Flatø, Muttarak & Pelser 2017:41–62). For instance, male farmers (17.10% and 71.2%) in Limpopo have more access to credit compared to their female counterparts (9.76% and 28.8%) (Mahasha 2019; Maziya et al. 2020:21–35). Hence, female farmers (54%) tend to be more vulnerable to climate variability compared to their male counterparts (46%) (Ubisi et al. 2017:27–38).

### Coping strategies of female farmers to climate variability

The main impacts of climate variability as observed by female farmers in Cameroon are changes in crop yields, prices and disposable incomes (Molua 2011:21–35). It has been reported that flooding events in northern Bangladesh and Nigeria caused female farmers to incur mass crop failures as their farms were submerged under water (Adeoti et al. 2016:1–16; Azad et al. 2013:190–199; Nnadi et al. 2019). Furthermore, Ezemonye (2015:109–116) reported that female farmers lost all their crops during the 2012 floods in Nigeria. In South Africa, Babugura et al. (2010) observed that female farmers have noticed significant losses in crop output and income because of low, unreliable rainfall patterns and increasing temperature.

However, farmers have developed strategies to cope with and mitigate the effects of climate variability. It has been reported that female farmers in Ghana and Pakistan borrow money from relatives, sell livestock and firewood, search for wild edible plants, temporarily migrate, engage in hunting and fishing and rely on remittances to cope with climate variability (Assan et al. 2018:86; Batool, Manzoor & Mahmood 2018:609–619). Furthermore, female farmers in Nigeria and the Eastern Himalayas changed harvesting dates and kept stocks which were fermented and sold to generate additional income (Adebo & Sekumade 2013:386–399; Singh et al. 2017:41–52). In South Africa, female farmers engage in nonagricultural activities and diversify their diets to cope with the effects of climate variability (Babugura et al. 2010; Ubisi et al. 2018:27–38).

In addition, female farmers in South Africa have limited access to diverse and cash-intensive coping strategies because of limited funding (Flatø et al. 2017:41–62). However, Alhassan et al. (2018:190–199) observed that female farmers in the northern region of Ghana used improved crop varieties, synthetic inputs (fertilisers and pesticides) and inorganic fertilisers (cash-intensive coping strategies) to cope with climate variability. It is evident from literature that female farmers in South Africa do not prefer soil conservation or cash-intensive coping strategies because of lack of capital, livelihood assets, technical knowhow (low literacy levels) and stringent labour requirements (Ajala 2017; Assan et al. 2018:86; Lawson et al. 2020:439–452). Despite these findings, there is currently limited information on the effects of climate variability on female smallholder farmers in South Africa; hence, more research is warranted.

## Methodology

### The research location

**FIGURE 1:**
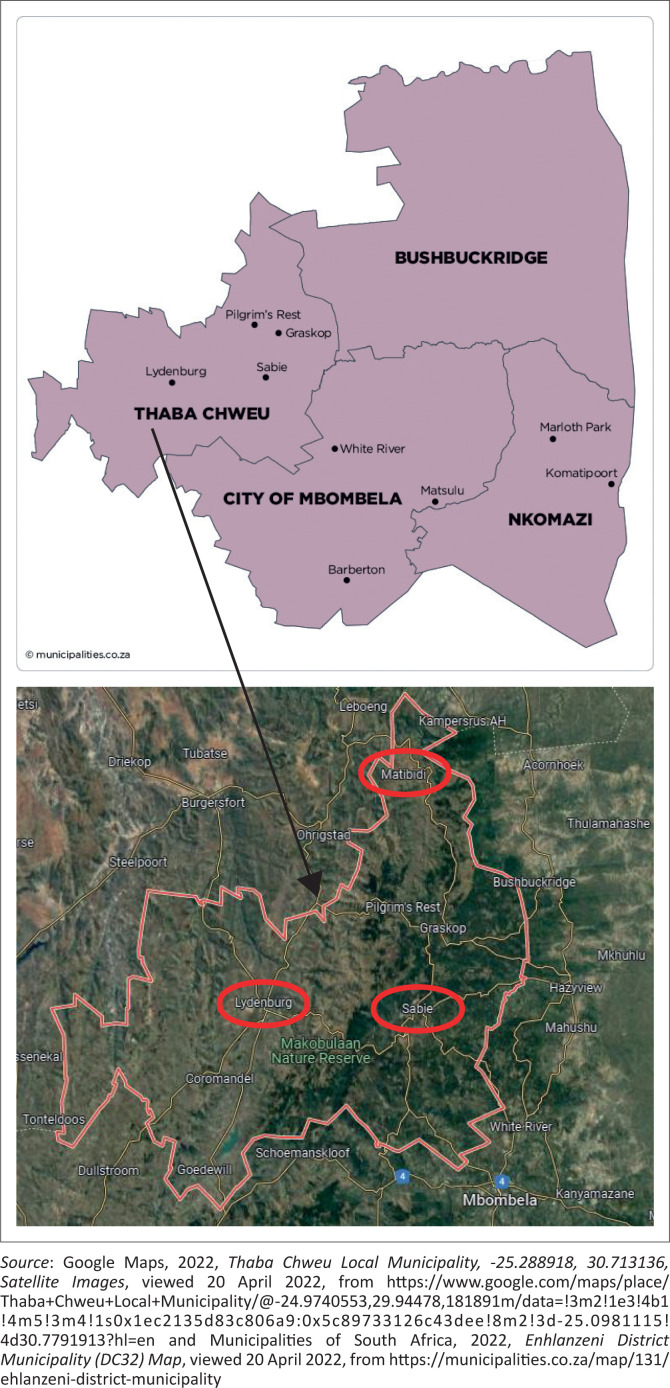
Ehlanzeni district.

Thaba Chweu Local Municipality is located in South Africa within the province of Mpumalanga. Thaba Chweu Local Municipality is a category B municipality that forms part of the Ehlanzeni District Municipality (Figure 1). The economy of TCLM is divided into three categories: urbanised, semi-urbanised and subsistence economies. The areas considered for this study are Lydenburg, Sabie and Matibidi. Thaba Chweu Local Municipality was chosen for our study because crop production has emerged as a vital livelihood activity for women and has been identified as a potential economic booster in this municipality. Furthermore, the municipality has experienced some climate variations over the years which have not been sufficiently studied.

### Research methodology

#### Research approach

This study adopted a mixed approach that integrated qualitative and quantitative methods. The qualitative approach allows for an extensive view and enhances the understanding of the scientific community regarding a particular phenomenon because the researcher is actively involved in the research process. This approach provided in-depth information on how vulnerable individual female farmers are to climate variability. The approach also examined the impacts on the farmers’ livelihoods, including strategies used to cope with fluctuating climatic conditions and their flexibility. On the other hand, quantitative research enables the researcher to mathematically analyse closed-ended questions. The use of a quantitative approach allowed for understanding the influence of demographic factors on perceptions and response practices of women to climatic risks.

#### Data collection procedure

The study used questionnaire-based, face-to-face interviews and online surveys to investigate the institutional, societal, cultural and economic constraints faced by female crop farmers. The interviews were also used to test the farmers’ knowledge about and experiences with climate variability, the factors which made them vulnerable to climate variability as well as their challenges and coping strategies. Despite the cost, face-to-face interviews were the most viable, as most of the farmers in the municipality are older, have low literacy levels and have limited access to the Internet. The face-to-face interviews observed COVID-19 protocols of wearing masks, physical distancing and hand sanitising. Where necessary, telephone-based interviews were used for the follow-up questions.

#### Sampling techniques and data analysis

Purposive, quota and snowball sampling techniques were used for this study. Adopting a combination of purposive, quota and snowball sampling techniques, the researchers targeted participants who were female farmers whose main livelihood activity was crop production. The desired sample size of 75 participants was reached through secondary referencing made by other chosen female farmers. The responses gathered were tabulated into thematic and quantitative data in Microsoft Excel spreadsheets for analysis of qualitative and quantitative data, respectively. In addition, data were presented through the use of graphs and tables and enriched by qualitative extracts of statements and views given by the participants.

### Ethical considerations

This serves to confirm the following regarding the article:

- It has not been simultaneously submitted to another journal for publication.
- The contribution of other authors has been properly acknowledged.
- No minors or children took part in the study.
- All adult interviewees were approached prior to interviews.
- The interviewees responded voluntarily in the study.
- A consent letter was signed before participants responded and there was no violation of human rights.
- Animals did not form part of the study.

The Department of Agriculture, Rural Development, Land and Environmental Affairs from the Mpumalanga province, South Africa approved this study.

## Results and discussion

### Demographic and socioeconomic characteristics

The demographic and socioeconomic characteristics of the respondents are summarised in Table 1. This study revealed that 92% of the respondents were breadwinners and were therefore responsible for meeting the primary needs of the household. Moreover, 65% of the farmers were aged 60 years and above. About 13% of the respondents had access to formal employment, whilst 84% were self-employed. The primary occupation of the self-employed group fell within crop production. Furthermore, 64% of the respondents had access to primary education, 19% to secondary education and 1% to tertiary education. A total of 16% had no access to education at all. These findings signify the low level of education in the municipality.

**TABLE 1:** Demographic and socioeconomic characteristics of female farmers in Thaba Chweu Local Municipality.

Characteristics	Frequency	Percentage (%)
**Are you a breadwinner?**		
Yes	69	92
No	6	8
**Total**	**75**	**100**
**Type of employment**		
Employed	10	13.33 (13)
Self-employed	63	84
Unemployed	1	1.33 (1)
Volunteering	1	1
**Total**	**75**	**100**
**Age**		
Less than 35 years	10	13
36–50 years	8	11
51–60 years	8	11
60 years and above	49	65
**Total**	**75**	**100**
**Race**		
Black	75	100
**Total**	**75**	**100**
**Level of education**		
No schooling	12	16
Primary schooling	48	64
Secondary schooling	14	19
Tertiary education	1	1
**Total**	**75**	**100**

*Source*: Primary data collected for this study, November 2020.

### Role of female farmers in crop production

**FIGURE 2:**
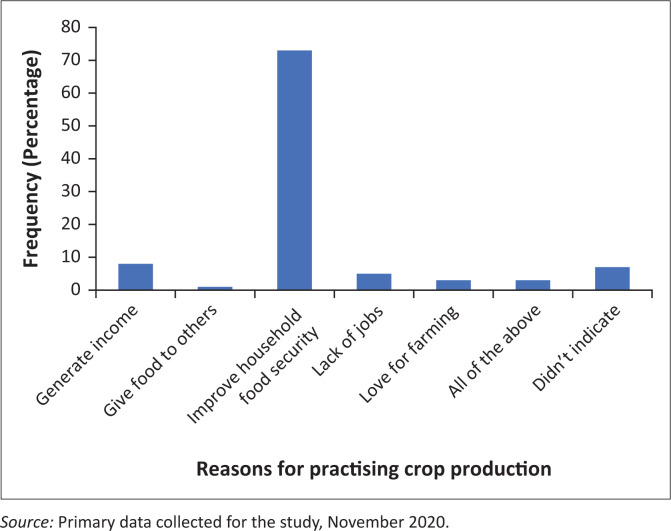
Reasons for practising crop production by female farmers in Thaba Chweu Local Municipality.

About 89% of the respondents indicated that they were responsible for soil preparation, fertiliser application, removing weeds, harvesting and crop sales. These findings are consistent with that of Maponya et al. (2013:273–282), who found out that female farmers in South Africa conduct most of the tasks on the farm such as ploughing the fields, removing weeds and monitoring crops and harvest dates. Furthermore, about 73% of the respondents stated that improving household food security was their main reason for practising crop production, as shown in Figure 2. This concurs with other studies that suggest that female farmers produce most of the food consumed across the globe and contribute positively to household calorie and dietary diversity (Adenugba & Raji-Mustapha 2013:51–58; Sraboni et al. 2014:11–52). Moreover, 8% of the respondents said their main reason for engaging in crop farming was to generate income, whilst a small number (5%) alluded to lack of alternative employment opportunities as their main reason for turning to farming. Galiè, Jiggins and Struik (2013:25–33) noted that changing economic conditions have made small-scale agriculture to be a less reliable source of income. This has caused men to engage in nonagricultural activities as women were left in charge of agricultural work. Galiè et al. (2017:1–8) revealed that women are more involved in farming more than their male counterparts and are therefore responsible for executing the majority of the tasks on the farm.

### Farming experience

The farming experience of female farmers with regards to crop production in the TCLM is summarised in Table 2. The majority (73%) of the respondents had crop farming experience of 20 years and above. This suggests that many of the farmers in the municipality are old and have ample farming experience. The most cultivated crops in the municipality were grain (69%) and spinach (12%), followed by cabbage, groundnuts, oyster mushrooms, pumpkin and tomato. Sixty-eight per cent of the respondents believed that the crop outputs have been fluctuating from one season to the other. Twenty-nine per cent of the respondents indicated that their crop outputs had declined over the years because of the decreasing rainfall and a much shorter growing season caused by the late onset of rainfall.

**TABLE 2:** Farmers’ experience and profile.

Item	Frequency	Percentage (%)
**Farming experience**		
Less than 5 years	7	9
6–10 years	4	5
11–20 years	9	12
20 years and above	55	73
**Total**	**75**	**100**
**Type of crop**		
All of the above (tomatoes, spinach and cabbage)	1	1
Cabbage	3	4
Grains	52	69
Groundnuts	3	4
Oyster mushrooms	2	3
Pumpkin	2	3
Spinach	9	12
Spinach and pumpkin	1	1
Tomato	2	3
**Total**	**75**	**100**
**Crop output (yields)**		
Decreasing	22	29
Increasing	2	3
Fluctuating	51	68
**Total**	**75**	**100**

*Source*: Primary data collected for the study.

### Challenges faced by female crop farmers

**FIGURE 3:**
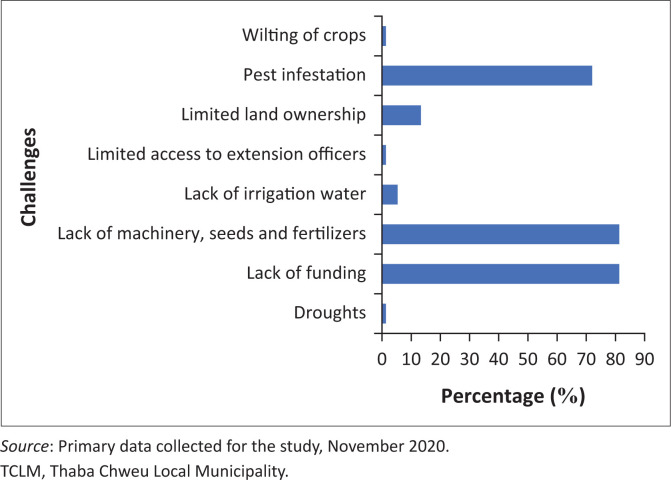
Challenges faced by female farmers in Thaba Chweu Local Municipality.

The main challenges faced by female farmers in the TCLM are lack of funding, farming equipment, seeds and fertilisers (81%), pest infestation (72%) and limited land ownership (13%) (Figure 3). This observation agrees with that of Ugwu (2019). These challenges have worsened the vulnerability of female farmers to the effects of climate change (Chandra et al. 2017:45–59). Similarly, Flatø et al. (2017:41–62) reported that female farmers have limited access to funding, which hinders them from procuring sufficient agricultural inputs, including limitations in accessing land and technology. As much as the post-1994 South African government has improved agriculture in the country, the respondents highlighted that they struggled to obtain tractors. Respondent 16, flabbergasted by limited tractors, remarked, ‘We have to halt tillage operations on the field and delay planting of our crops because we do not have money to hire a tractor’. The farmers are expected to pay a fee because most of the tractors are owned by private organisations. This situation hinders them from carrying out proper tillage operations, leading to compromised crop yields and productivity. Furthermore, respondent 25, commenting on the challenge of limited funding, also remarked, ‘We cannot stop livestock invasion on farming plots as we do not have money to buy and erect a fence’.

It was reported that female land ownership patterns vary from one geographical area to the other in the municipality. A majority of the respondents in the community of Matibidi have unlimited access to land. However, the land ownership rights of the respondents are tied to their male counterparts as per the traditional system. This contradicts the findings by Cousins (2013:73–99), who reported that the traditional leaders of the Mchunu Council, KwaZulu-Natal also allocated land to single women (Cousins 2013:73–99). The challenge of limited access to land in the study area prevented the respondents from diversifying crops and receiving help from the municipality, as the majority of the land is owned by private individuals. Respondent 20 expressed that ‘I cannot diversify crops and receive help from the municipality because the land we plant on is owned by private individuals’. This implies that female farmers do not have security of tenure. These findings are similar to a study which reported that the traditional leaders in the Matsamo Traditional Authority made a decision to end subsistence cultivation (practised by female farmers) on communal land without consulting female farmers (Rangan & Gilmartin 2002:633–658). The existing legislations, such as the Communal Land Rights Act of 2004, the Restitution Act, the Community Property Association and the Land Reform Pilot Programme, which were designed to enable women to make independent land claims and to be members of tenure decision-making bodies, have not improved female land ownership rights in South Africa (Cousins 2013:73–99; Meer 1997:133–144). The challenges with the land reform policies and programmes are limited participation of women in land allocation decision-making bodies, placement of land under traditional leaders who tend to prioritise men over women and lack of emphasis on the impact of gender relations in land ownership patterns in South Africa. Hence, the newly formed LEWC policy has been heavily criticised because of its lack of emphasis on the impact of gender relations on land ownership patterns in South Africa (Mubecua & Nojiyaza 2019:7–19).

The effects of the challenges as identified by the respondents (Figure 3) were inability to procure seeds owing to lack of funding (76%), reduced yields (4%), loss of revenue (1%) and delayed planting (1%). Similar effects were reported by Molua (2011:21–35) who indicated that female farmers were susceptible to climate related risks due to being over dependent on climate sensitive livelihoods.

### Mitigation measures

**FIGURE 4:**
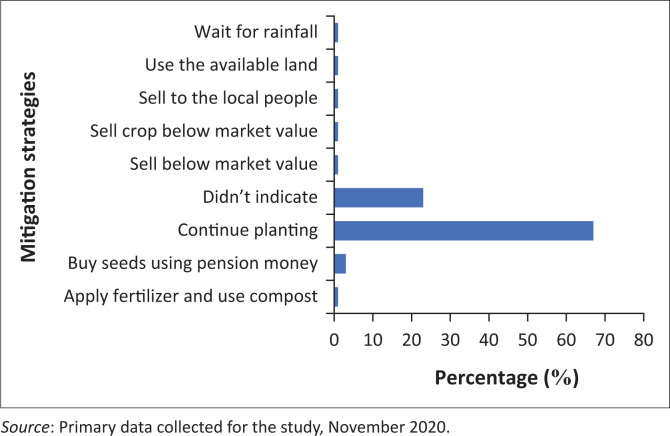
Strategies to mitigate the effects of climate risks in Thaba Chweu Local Municipality.

Sixty-seven per cent of the respondents indicated that they practice continuous planting as a measure to mitigate the effects of the challenges (Figure 4). However, 23% of respondents indicated that they do not take any measures to respond to the challenges. In addition, 3% of the respondents indicated that they use their money to buy seeds, apply fertilisers and make use of compost, whilst 1% of the respondents have resorted to selling their crops below market value, selling to the residents or simply waiting for rainfall. Therefore, it can be suggested that the majority of the female farmers in TCLM are highly vulnerable as they do not implement appropriate climate response mechanisms. This limitation can be attributed to low education levels, lack of adequate resources (funding, agricultural inputs) and lack of economic opportunities in the municipality.

### Female farmers’ perceptions about climate change

There were varied perceptions and understandings of climate variability amongst the participants (Table 3). With regards to how the participants perceive changes in their local environment, 99% indicated that the climate conditions have changed. About 79% mentioned that the rainfall levels have been on the decline over the years, whilst 13% indicated that temperature levels have been increasing. The most noticeable form of environmental change included droughts and strong winds (67%), followed by high temperatures (16%), droughts (9%), heavy rain (4%) and delayed rainfall (4%). This supports the findings of Dab-gbeto and Villamor (2016:297–308), who reported that the majority of the female farmers in Nigeria perceive climate variability in terms of droughts and strong winds, and Singh et al. (2017:41–52), who also reported that female farmers in the Eastern Himalayas perceive climate variability in terms of reduction in rainfall, rising temperatures and droughts.

**TABLE 3:** Farmers’ perceptions and understanding of climate variability.

Item	Frequency	Percentage (%)
**Have the climatic conditions in your area changed?**		
Yes	74	99
No	1	1
**Indicators of changes in climatic conditions**		
Did not indicate	1	1
Rainfall and temperature vary according to season	5	7
Rainfall is decreasing	59	79
Temperature is increasing	10	13
**Types of environmental change**		
Droughts	7	9
Droughts and strong winds	50	67
Heavy rainfall	3	4
High temperature	12	16
Delayed rainfall	3	4
**Causes of climate variability**		
Ancestors	5	7
Ancestors and human activities	8	11
Cultural reasons	2	3
Did not indicate	1	1
God and nature	1	1
Human activities	8	11
Nature	50	67

*Source*: Primary data collected for the study, November 2020.

Furthermore, 67% of the respondents said that changes in climatic conditions were a result of natural causes, whilst 11% believed that changes in climatic conditions were caused by human activities. In addition, 7% and 3% of respondents believed that ancestral and cultural reasons contributed to changes in climate conditions, respectively. Respondent 40, commenting on the cause of climate variability, remarked, ‘Our ignorance and negligence of the ancestors’ ways of living and doing things is the cause of decreasing rainfall levels and increasing temperatures’. This agrees with a study by Lolig et al. (2014:542–553), who reported that female farmers in Ghana use ancestral worship to cope with climate variability as they believe that climate variability is a punishment from the gods. The findings of the study indicate that a majority of the female farmers in TCLM have well-informed beliefs regarding the causes of climate variability despite low literacy levels and old age.

### Effects of climate variability

Ninety-five per cent of the respondents indicated that the main effects of climate variability are low crop outputs, followed by food insecurity (77%) and loss of disposable income (16%). This is in line with the findings of Molua (2011:21–35), who highlighted that the biggest effects of climate variability are changes in crop yields, food security and disposable income. Low crop outputs are attributed to decreased rainfall and high temperature, which shortens the growing season and leads to increased losses in crop and seed stocks as a result of low crop outputs and delayed planting (Derbile et al. 2016:1–9). The crops that have shown significant decline in yields are cabbage, lettuce, grains and pumpkin. Respondent 40, commenting on the effects of climate variability on crop outputs, remarked, ‘There was a year I harvested 54 bags of grains and I have not been able to replicate these yields in subsequent years’. Loss in crop outputs results in food insecurity, as a majority of the farmers in the municipality engage in crop production as a source of livelihood. In South Africa, female farmers have noticed massive crop losses and reduction in food accessibility because of unreliable rainfall patterns and increasing temperatures (Babugura et al. 2010; Ubisi et al. 2017:27–38). Elsewhere, female farmers in Nigeria lost a significant share of their incomes during 2012 floods (Ezemonye 2015:109–116). In this study, 16% of the respondents identified loss of income as an effect of climate variability. The participants attributed this to inferior yields and loss of crop quality as a result of lack of irrigation systems, greenhouses and agricultural inputs (drought-resistant seeds, fertilisers and pesticides). Poor crop quality has a negative effect on the income levels of the farmers, as they would be forced to sell below market value and thus incur financial losses. In addition, lack of sufficient produce to sell because of low crop yields can also be attributed to loss of household income.

## Financial assistance for crop related loss

A majority (92%) of the respondents indicated that they do not receive any form of financial assistance for crop failure, whilst 8% of the farmers highlighted that they receive assistance from the government. Furthermore, 5% of the respondents said they relied on family members for financial support. Earlier studies reported that female farmers in South Africa tend to receive less state support compared to their male counterparts (Hart & Aliber 2012:48). Female farmers do not receive sufficient support to participate in revenue-generating activities because of limited resources and cultural ideologies that promote the superiority of men, as well as skewed land ownership patterns (Monwar, Hossen & Kalam 2018:125–136). Moreover, women may not receive any form of assistance because crop production is culturally regarded as an activity for men, whereas women are deemed as being unproductive farmers (Myeni et al. 2019:3003). In addition, female farmers have limitations in accessing financial assistance from formal institutions (banks) because of lack of collateral and inability to afford the high transaction costs associated with borrowing credit (Ugwu 2019). Therefore, it has been recommended that government should establish banks and credit facilities in rural areas to enable smallholder farmers to have access to credit (Kemi 2017:102–106). Regarding the state support, there seems to be a mismatch with the type of support offered by the municipality and expectations of the farmers. The resident extension officer highlighted that the female farmers expect the government to provide all the basic inputs on an annual basis free of charge. On the other hand, the government expects the farmers to become independent and sustainable by improving their crop sales and procuring their own agricultural inputs. Therefore, it is imperative for the government to organise workshops to explain their role to the farmers and train them to become viable and sustainable (generate sufficient income and establish sustainable farming enterprises).

## Coping Strategies

### Types of coping strategies

**FIGURE 5:**
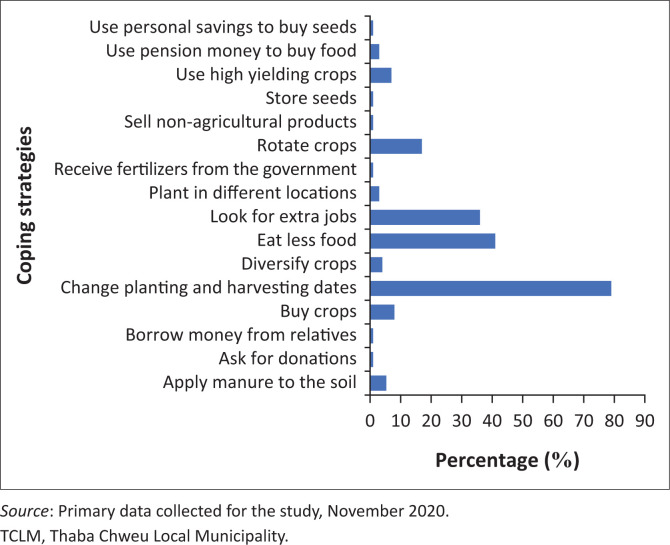
Coping strategies to climate variability and socio-economic challenges adopted by female farmers.

As a first coping strategy to climate variability and socio-economic challenges (Figure 5), 79% of the respondents said they change planting and harvesting dates. The second and third most preferred coping strategies were eating less food (41%) and looking for extra jobs (36%), respectively. The fourth most preferred coping strategy was crop rotation (17%). The least preferred coping strategies included the use of high-yielding crops, application of manure and compost to the soil, purchasing crops from supermarkets, asking for donations, receiving money from the government (pension) and using personal savings to buy seeds. This demonstrates that most female farmers in TCLM still used insecure coping strategies. Thus, the majority of the respondents preferred eating less food as a strategy to cope with climate variability. Some of the respondents, however, engaged in nonagricultural activities and used pension money to supplement food shortages caused by crop failures. Respondent 51, commenting on the strategies used to cope with climate variability, remarked, ‘I sell agricultural and nonagricultural products in the market to cover food shortages at home’. This is consistent with the findings of Babugura et al. (2010), who found that some female farmers in South Africa engaged in nonagricultural activities to generate additional income and supplement food shortages caused by climate variations.

#### Determinants of coping strategies

Gender, livelihood assets, age, education, marital status, preferences, engaging in off-farm activities, land ownership and regular access to extension services determined the type of coping strategies that women would adopt (Alhassan et al. 2017:190–199; Lawson et al. 2020:439–452). In this study, lack of machinery, seeds and fertilisers (73%) were the leading determinants of the coping strategies adopted by the female farmers. Second was farming experience (13%), followed by lack of funding (4%). Limited access to funding (4%), soil type (4%) and limited access to agricultural information (1%) were also reported as having an influence on coping strategies. Atsiaya et al. (2019:1–12) reported that 9% – 12% of the women in Kenya had access to secondary and tertiary education. The lack of tertiary education prohibits women from adopting innovative coping strategies such as irrigation systems (Batool et al. 2018:609–619). Despite limited access to tertiary education, women have used their indigenous knowledge and skills to implement agricultural practices which enable them to cope with variations in climatic conditions (Abeka et al. 2012:8–51).

#### Effectiveness of coping strategies

The effectiveness of the coping strategies adopted by the respondents in TCLM is summarised in Table 4. About 71% of the respondents indicated that the coping strategies did not improve yields, owing to the shortening growing season and their inability to cope with high temperatures. Respondent 60, commenting on the effectiveness of the coping strategies, expressed that ‘there is nothing we can do to stop the increasing temperatures’. This implies that female farmers in the TCLM do not have access to greenhouses and irrigation systems, which are technologies that can be used to mitigate the effect of high temperatures on crop productivity. Furthermore, the farmers in the community of Matibidi could not erect a fence around their farms to prevent livestock-related crop losses because of the lack of funding. However, 35% of the respondents indicated that the coping strategies have improved crop quality, crop yields, food security, sales, soil health and limited pest infestation. Crop rotation improves soil health, which results in improved crop quality, yields and food security. The findings of the study demonstrate that the lack of greenhouse and irrigation systems reduces the effectiveness of the coping strategies adopted by the female farmers. Therefore, the municipality should support the farmers with greenhouses and irrigation systems because of decreasing rainfall levels in most areas of the municipality.

**TABLE 4:** Effectiveness of coping strategies on crop productivity.

Effects	Frequency	Percentage (%)
**Negative**		
Yields have not improved	51	68
Cannot stop hot temperatures	2	3
**Positive**		
Improves yields	13	17
Improves crop quality	4	5
Improves sales and revenue	3	4
Improves food security	2	3
Prevents crop failure	2	3
Soil health and pest infestation	2	3

*Source*: Primary data collected for the study, November 2020.

## Conclusion

This article aimed to provide an empirical report on the key challenges faced by female crop farmers in the TCLM. The findings of the study demonstrated that female farmers in the municipality were vulnerable to climate variability because of lack of funding, limited access to funding, agricultural inputs and limited land ownership. These barriers have caused the female farmers to grapple with reduced crop yields, income and food insecurity and adopt unsustainable, conservative and capital-intensive coping strategies. The coping strategies adopted by the farmers include changing planting and harvesting dates, crop rotation, reduced food consumption and engaging in nonagricultural activities. However, the majority of these coping strategies adopted by the female farmers had little impact on their livelihoods, food security and crop productivity (yield), owing to limited access to drought-resistant crops, greenhouse and irrigation systems and sluggish economic growth in the municipality over the past few years (closure of industries). In addition, the study also found that a majority of the female farmers did not receive any form of assistance for crop-related losses. However, farmers got some form of assistance from the state in the form of an agricultural extension officer and agricultural inputs, even though they deem it to be inadequate. This demonstrates that, inasmuch as the government has developed interventions to assist small-scale farmers, the programmes are not gender-sensitive and not adequate to address the unique challenges experienced by women.

This study recommends that government and the local municipal officials should constantly conduct field visits to assess the efficacy of their programmes. Furthermore, the government and local municipal officials should also organise sessions where farmers are taught about the role of state, because there seems to be a misconception amongst the farmers with regards to the type of support they should get from the state. In addition, the government and local municipal officials should also organise marketing workshops to train the farmers on marketing and other income-generating projects to become commercially viable farmers. There is also need to incentivise young people who engage in agricultural activities for the purpose of attracting them into the farming sector.
